# Cellular Heterogeneity of the Heart

**DOI:** 10.3389/fcvm.2022.868466

**Published:** 2022-04-25

**Authors:** Nathaly Anto Michel, Senka Ljubojevic-Holzer, Heiko Bugger, Andreas Zirlik

**Affiliations:** University Heart Center Graz, Department of Cardiology, Medical University of Graz, Graz, Austria

**Keywords:** single cell RNA sequencing, heart, cell type, heterogeneity, genes

## Abstract

Recent advances in technology such as the introduction of high throughput multidimensional tools like single cell sequencing help to characterize the cellular composition of the human heart. The diversity of cell types that has been uncovered by such approaches is by far greater than ever expected before. Accurate identification of the cellular variety and dynamics will not only facilitate a much deeper understanding of cardiac physiology but also provide important insights into mechanisms underlying its pathological transformation. Distinct cellular patterns of cardiac cell clusters may allow differentiation between a healthy heart and a sick heart while potentially predicting future disease at much earlier stages than currently possible. These advances have already extensively improved and will ultimately revolutionize our knowledge of the mechanisms underlying cardiovascular disease as such. In this review, we will provide an overview of the cells present in the human and rodent heart as well as genes that may be used for their identification.

## Introduction

Cardiovascular disease (CVD) and its sequelae represent a major health and socioeconomic burden accounting for vast and continuously increasing morbidity, and roughly a third of all deaths in the world (1). There are many different types of CVD including coronary heart disease, stroke, peripheral arterial disease, myocardial disease, and aortic disease. Coronary heart disease can lead to angina, heart attacks, or heart failure. Although the exact cause of CVD is unknown, a solid body of experimental and clinical data identified inflammation as a common final pathomechanism. This is particularly well documented for atherosclerosis and its direct clinical consequences (2), however, increasing evidence suggests that traditional and non-traditional risk factors trigger this inflammatory process and thus ultimately drive initiation and progression of CVD (3, 4). These risk factors include smoking, hypertension, hypercholesterolemia, chronic kidney disease, and diabetes, but also systemic inflammation stemming from chronic inflammatory conditions (e.g., rheumatoid arthritis), infectious diseases, or obesity-derived visceral adipose tissue inflammation. However, how this pathogenic link between (multiple) risk factors, inflammation, and adverse cardiac phenotypes operates, and which cellular phenotypes or clusters mediate its action in the heart is largely unknown.

Identification of cellular heterogeneity and their intercommunication can play a vital role in differentiating a healthy from a diseased heart and it may predict future outcomes with superior precision and at much earlier stages than currently possible. Conventional ways used to identify specific cells include fluorescence-activated cell sorting (FACS). FACS is a powerful tool that allows simultaneous multiparametric analysis of the physical and chemical characteristics of up to thousands of particles per second. Nonetheless, FACS affords some limitations in sample preparation, fluorescent parameters, antibodies selection and most importantly, paucity of markers that can be assayed at the same time. Therefore, FACS is not suited for an unbiased identification and further subclassification of unknown or poorly subcategorized cells and the definition of their potential roles in physiology and disease pathology.

In the last 10 years, analyses of cellular heterogeneity with single cell resolution have made an astounding progress. Developments in high parametric multiplex cell analysis such as cytometry by time of flight (CyToF) and single cell RNA sequencing (scRNAseq) have enabled us to gain a high-power view on novel individual cellular phenotypes as well as on distinct cellular expression patterns integrating the transcripts of thousands of genes (5). While such high-parametric data sets are still scarce in cardiovascular disease, scRNAseq techniques have been successfully employed to detail the cellular composition of whole organs, to identify new cell types, and/or characterize cellular expression patterns associating with disease, disease severity, outcome, or therapeutic response in other areas (6–10). Therefore, they have proven to be an excellent tool to advance our understanding of the cell types and populations involved in disease pathogenesis in various fields. Such knowledge is urgently needed to better understand cardiac physiology and its derangement through disease and to ultimately improve the treatment options and outcomes for cardiac patients.

**FIGURE 1:**
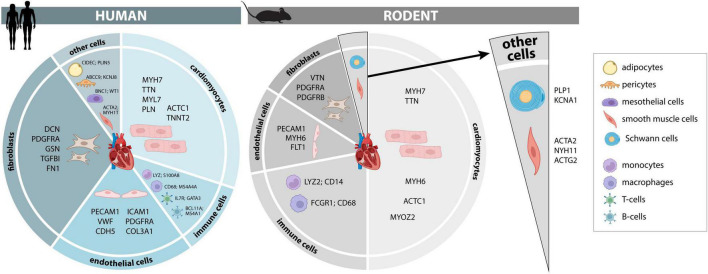
Cellular composition and markers of the human and murine heart. Cellular composition and most consistently identified markers that may be used for identification and separation of major cellular lineages in adult human and murine hearts. A more comprehensive list of additional marker genes, including fetal human hearts, is provided in Supplementary Tables 1–3.

In the following sections, we summarize current knowledge on the cellular composition of the heart, its relevance for physiology and pathologic transformation, as well as characteristic changes in pathological conditions reported. We also provide a comprehensive overview of the developmental stage-specific changes in cellular heterogeneity and most promising cellular markers that can increase robustness and reproducibility of single cell transcriptomic analyses in different experimental animal models and human biomaterials (Figure 1 and Supplementary Tables 1–3). Given that methodological differences between scRNAseq and FACS complicate data interpretation and would require a large discussion, we predominantly focus on publications reporting data generated by scRNAseq.

## Cellular Composition of the Heart

### Cardiomyocytes – The Core of Cardiac Contraction

Cardiomyocytes are the engine of the heart where energy is converted to mechanical work by myosin ATPases consuming high energy phosphates, thereby driving the cross-bridge cycle of the cardiomyocyte. While the use of stereological and morphometric methods estimated that cardiomyocytes would cover 75% of the total cardiac cell volume in rats, studies based on flow cytometry and immunohistochemistry revealed that around 30% of total cardiac cells are cardiomyocytes, with significant species differences in the cellular composition between human, mouse and rat hearts (11–13). Confident identification of cardiomyocytes can be achieved by measuring the expression proteins involved in the contractile machinery such as myosin light (*MYL2-4, MYL7*, and *MYL9*) and heavy (*MYH6, MYH7*, and *MYH7B*) chains, myosin binding proteins (*MYBPC3*), troponins (*TNNT2, TNNTI1, TNNTI3*, and *TNNTC1*), and proteins involved in calcium-mediated processes [ryanodine receptor 2 (*RYR2*), phospholamban (*PLN*), Sodium ion/calcium exchanger (NCX)] (14–16). Integrity of cardiomyocytes is reflected by expression of the protocadherin 7 (PCDH7) gene which encodes a strong calcium-dependent adhesive molecule (17), and of SET (Suppressor of variegation, Enhancer of Zeste, Trithorax) and MYND (Myeloid-Nervy-DEAF1) domain containing 2 (*SMYD2*) corresponding to a lysine methyltransferase that promotes sarcomere formation and stabilization (18). It seems that the latter proteins are only significantly expressed in cardiomyocytes of the adult heart.

Among regularly contracting cardiomyocytes, atrial and ventricular cardiomyocytes participating in the cardiac conduction system can be distinguished which transduce electrical stimuli to drive cardiac contraction. These cardiomyocytes have been traditionally identified based on hyperpolarization-activated cyclic nucleotide-gated potassium channel 4 (*HCN4*) (19) and contactin-2 (*CNTN2*) expression (20). Additional markers include gap junction protein alpha-1 (*GJA1*), gap junction protein alpha-5 (*GJA5*), secreted protein acidic and cysteine rich (SPARC)-related modular calcium-binding protein 2 (*SMOC2*), ISL LIM homeobox 1 (*ISL1*) or T-box transcription factor 3 (*TBX3*), although the expression of these markers shows significant heterogeneity depending on their anatomical location within the conduction system (nodes, atrioventricular bundle, Purkinje fiber network) (21–23). Using scRNAseq technology, an even higher grade of heterogeneity within the conduction system has been revealed. Specific subpopulations of cardiomyocytes with particular expression of myozenin 2 (*MYOZ2*) in adult murine and developing human heart (24) have been identified, and the expression of insulin-like growth factor-binding protein 5 (*IGFBP5*), copine 5 (*CPNE5*), and neurotrimin (25) is enriched in the entire conduction system whereas *SMOC2* expression is exclusively observed in cells of the sinus node (26).

To distinguish the fetal and adult heart, *TNNTI1*, *TNNTI3*, and *TNNTC1* are accepted cellular markers in fetuses, whereas different troponin and myosin genes are commonly used to identify adult cardiomyocytes (*TTN, MYBPC3, TNNT2, MYH7*, and *MYL2*) (14–16). Of note, while the known switch toward predominant expression of *MYH6* in postnatal hearts (compared to predominant expression of *MYH7* in fetal hearts) is observed in rodents, humans mainly express *MYH7* throughout life without a significant isoform switch. Furthermore, α-skeletal actin (*ACTA1*) is mainly expressed in fetal and neonatal human hearts, whereas expression of *ACTC1* predominates in the adult heart (27, 28). Fetal hearts also show enhanced expression of compliant titin (*TTN*) isoforms (N2BA1/N2BA2), which are eventually replaced by adult isoforms in postnatal development (29).

A differentiation between fetal and adult cardiomyocytes can also be achieved by evaluating markers of cell proliferation, given the high and low proliferative capacity of fetal and adult cardiomyocytes, respectively. Convenient markers to appreciate cardiomyocyte proliferation may include expression levels of DNA topoisomerase 2 alpha (*TOP2A*) and marker of proliferation Ki-67 (*MKI67*) (14, 30, 31). It remains to be noted that only few cardiomyocytes in the adult heart (0.4% of all cardiac cells) display markers of proliferation at all as revealed by an integrative analysis using a mouse cell type atlas in combination with single-nuclei RNA-seq (32).

Furthermore, a differentiation between fetal and postnatal cardiomyocytes is also possible by measuring markers or metabolites of energy metabolism (33). While fetal hearts mainly utilize carbohydrates for ATP regeneration and have low mitochondrial oxidative capacity, cardiomyocytes switch toward predominant fatty acid oxidation and undergo marked mitochondrial biogenesis once fatty acid and oxygen availability increase in the early postnatal period (34). The fetal metabolic program is characterized by expression of the liver isoform of carnitine palmitoyltransferase 1 (*CPT1*), whereas human postnatal cardiomyocytes express muscle *CPT1*, high levels of peroxisome proliferator-activated receptor alpha (*PPAR*α; induced by fatty acid availability) and *PPAR* gamma coactivator 1-alpha (*PGC-1*α; driving physiological mitochondrial biogenesis). In addition, the content of glycogen (measurable by histology are electron microscopy) may be higher than 30% in fetal but only around 2% in adult cardiomyocytes (33).

Atrial and ventricular myocardium differs in developmental, structural, hemodynamic, and physiological properties, which is reflected by a distinct expression profile (35). Based on these differences, Hairy/enhancer-of-split related family bHLH transcription factor with YRPW motif 2 (*HEY2*) and *MYH7* may qualify as markers predominantly expressed in ventricular cardiomyocytes, whereas expression of natriuretic peptide A (*NPPA*) and *MYL4* is more evident in atrial cardiomyocytes (16). Furthermore, the atrial fibrillation susceptibility gene, paired like homeodomain 2 (*PITX2*), is only observed in left atrial cardiomyocytes (36). Interestingly, *HCN4* which encodes the ion channel responsible for spontaneous depolarization and which has also been associated with atrial fibrillation, is present in approximately 4% of right atrial, in less than 0.5% of left atrial and only ∼1% of ventricular cardiomyocytes (16).

In non-diseased myocardium, a significant heterogeneity of cardiomyocytes with gradients of specific gene expression has been observed, and additional changes in gene expression are superimposed by cardiac pathologies, complicating the interpretation of transcriptional profiling studies on the single cell level. Application of single-cell technology has revealed a more extensive heterogenic gene expression (e.g., *NPPA*; brain natriuretic peptide, *BNP*; MYH7) in failing hearts than previously identified using bulk-RNA sequencing (37). In another study, the combination of single-cell analysis and RNA *in situ* hybridization of human dilated cardiomyopathy samples uncovered transcriptional heterogeneity, allowed to distinguish distinct gene modules responsible for cardiomyocyte hypertrophy or failure, and elucidated coordinated molecular and morphological dynamics of cardiomyocytes that may promote heart failure development (38). Furthermore, studies in rodents with pressure overload-induced cardiac hypertrophy showed that *MYH7* genes were greatly expressed with smaller cardiomyocytes as opposed to larger cardiomyocytes, and that *MYH7* was markedly expressed in middle layers of the myocardium (38). These data support the concept of adaptive heterogeneity of cardiomyocytes, where cardiomyocytes that consume less energy undergo atrophy and express major histocompatibility complex (MHC) proteins to limit myofibrillar ATPase activity, whereas other cardiomyocytes with increased oxidative capacity may express MHC proteins to guarantee a high rate of myofibrillar ATP consumption Using these novel technologies to provide a spatial map of cells-of-interest within the heart or to identify cardiomyocytes of interest may have intriguing clinical value, e.g., by facilitating diagnostics of myocardial tissue or biopsy specimen by pathologists.

Cardiomyocytes are the main cellular population responsible for cardiac contraction, and their unique molecular composition allows them to accomplish their highly specialized roles. However, overall cardiac function needs to be investigated and understood in the context of cardiac tissue, in which other cell types operate together with cardiomyocytes to orchestrate periodic cardiac contractions that adapt to the physiological demands of the living system.

### Fibroblasts – Frequent and Functional

Cardiac fibroblasts are involved in the synthesis and remodeling of extracellular matrix, communicate with cells of the immune system, participate in cardiac conductivity and rhythmicity, and take part in myocardial healing responses, e.g., following myocardial infarction or during chronic disease states (39–41). In absence of disease, fibroblasts are nearly equivalent to cardiomyocytes in cell number, accounting for approximately 25–32% of all cells in the heart (16, 42). In adult human heart tissue, classical fibroblast markers include decorin (*DCN*), gelsolin (*GSN*), transgelin (*TAGLN*), regulator of G protein signaling 5 (*RGS5*), Smooth muscle aortic alpha-actin 2 (*ACTA2*), Thy-1 cell surface antigen (*THY1.1*), platelet derived growth factor receptor alpha (*PDGFRA*), S100 calcium binding protein A4 (*S100A4*), discoidin domain receptor tyrosine kinase 2 (*DDR2*), lymphocyte antigen 6 complex, locus A (*LY6A*) also known as Sca-1, vimentin (*VIM*), and collagen type I alpha 1 chain (*COL1A1*) (41). However, once fibroblasts become activated or the heart suffers injury (e.g., myocardial infarction), fibroblasts may also strongly express other markers such as periostin (*POSTN*), alpha smooth muscle actin (α*SMA*) or mesenchyme homeobox 1 (*MEOX1*), among other genetic signatures. In this respect, a differentiation of three major types of fibroblasts in the heart by distinct expression profiles (mature fibroblasts, activated fibroblasts, myofibroblasts) has been provided in another excellent review (41). Fibroblasts of the fetal human heart express transcription factor 21 (*TCF21*), smooth muscle cells snail family transcriptional repressor 2 (*SNAI2*), *COL1A1*, collagen type I alpha 2 chain (*COL1A2*), *DCN*, delta like non-canonical Notch ligand 1 (*DLK1*), and lumican (*LUM*). Similar to cardiomyocytes, fibroblasts also exhibit differential expression between atria and ventricles, and between the left and right heart. Good examples are cartilage intermediate layer protein 2 (*CILP*) and integrin beta-like 1 (*ITGBL1*) which are upregulated in the left ventricle while downregulated in the right ventricle (41). Human and rodent genes involved in pathological remodeling of the heart or considered as profibrotic markers are cytoskeleton associated protein 4 (*CKAP4*), NADPH oxidase 4 (*NOX4*), insulin like growth factor 1 (*IGF1*), A disintegrin and metalloproteinase (ADAM) with thrombospondin type 1 motif 4 (*ADAMTS4*), vascular cell adhesion molecule (*VCAM*), and AXL receptor tyrosine kinase (*AXL*) (16, 24, 43, 44). For example, in the event of myocardial ischemia reperfusion, an increase in the expression of *CKAP4* was identified in activated fibroblasts (24), a protein considered also responsible for development of atrial fibrosis in the heart (45). Interestingly, myocardial biopsies of patients suffering from ischemic heart disease also show increased expression of *CKAP4* in fibroblasts, accompanied by activation of other genes such as *POSTN*, WNT1-inducible signaling pathway protein-1 (*WISP1*), and tenascin C (*TNC*) (24) as well AE binding protein 1 (*AEBP1*) a novel transcription factor identified in human cardiac fibrosis (46).

In mice, fibroblasts have been intensely studied. In young mice, fibroblasts constitute 15–19% of the healthy murine heart, whereas in adult mice, this percentage increases to more than 20% (47). Characteristic markers used to identify fibroblasts in murine hearts include *COL1A1*, *GSN*, *DCN*, WNT inhibitory factor 1 (*WIF1*), dickkopf WNT signaling pathway inhibitor 3 (*DKK3*), metallothionein 2 (*MT2*), TIMP metallopeptidase inhibitor 1 (*TIMP1*), *PDGFRA*, and *TCF21* (48). In the context of disease, expression of the fibrosis-associated extracellular matrix genes, *POSTN* and fibrillin 1 (*FBN1*), are increased in a mouse model of pediatric mitochondrial cardiomyopathy, although expression of these genes has been reported in other cell types as well (47). Upon ischemia reperfusion, fibroblasts show high expression of *POSTN*, *WISP1*, and *TNC*, associated with fibroblast activation (24). Another study in mice demonstrated that gene expression of fibroblasts was skewed toward Ki-67, *COL1A2*, collagen type III alpha 1 chain (*COL3A1*), collagen type V alpha 1 chain (*COL5A1*), *SPARC*, secreted frizzled related protein 2 (*SFRP2*), and *DKK3* following myocardial infarction (49). However, in pigs the infarct zone presented upregulation of ACTA2, COL1A1, TIMP2, POSTN, TAGLN, MMP2, and FN1 genes specifically in the infarct zone (50).

Aging has a significant impact on the fibroblast expression profile and function. Comparing transcriptomes of 12 week-old and 18 month-old mouse hearts using single-nucleus RNA Seq revealed that aging predominantly affected fibroblast gene expression, and a total of 12 age-dependent fibroblast subclusters were identified (51). Gene ontology analysis of differentially regulated genes elucidated that aging predominantly affected expression of genes related to inflammatory/immune responses, extracellular matrix organization, angiogenesis, and osteogenesis. In particular, expression of serine protease inhibitors (SERPIN) family E member 1 and 2 (*SERPINE1* and *2*) was increased in certain fibroblast clusters, promoting antiangiogenic effects upon their secretion. Furthermore, some fibroblast subclusters identified in this study showed higher expression of genes involved in osteoblast differentiation such as *DDR2*, runt related transcription factor 2 (*RUNX2*), glycoprotein M6B (*GPM6B*), JunB proto-oncogene, AP-1 transcription factor subunit (*JUNB*), and CCAAT enhancer binding protein beta (*CEBPB*), showing a transition toward an osteogenic fate. This osteogenic transition seems to be particularly evident within epicardial layers of the aged heart (51).

In a study focused specifically on the role of active fibroblasts in myocardial infarction in mice, a subpopulation called cells reparative cardiac fibroblasts (CFRs), was identified by the expression of POSTN and collagen triple helix repeat containing 1 (*CTHRC1*). CFR activity appears to be essential in scar healing following MI (52). Of note, the CFR signature was also found in MI model in swine, and at least in part in human myocardial biopsy specimen taken from the ischemic zone of ischemic cardiomyopathy or from dilated cardiomyopathy, indicating this signature may be conserved across species and highlighting the translational significance of these findings.

Interestingly, specifically targeting fibroblast may reduce fibrosis in mice suffering myocardial injury (53, 54). Aghajanian et al. set out to identify proteins specifically expressed by activated cardiac fibroblasts in a MI mouse model. They found that fibroblast activation protein alpha (FAP) is one of the main responsible for fibrosis and that FAP was also previously observed in human and rat hearts after MI (55). Of note, targeting FAP by nanoparticle-mediated generation of chimeric antigen receptor (CAR) T cells, *in vivo* resulted in a reduction in cardiac fibrosis suggesting that this approach may hold promise for treatment of fibrosis in cardiac disease states (56, 57).

Approximately three decades ago, fibroblasts were suggested to be considered as immune cells because of their ability to produce cytokines when stimulated with IL1 or TNFα. They are also capable of producing prostaglandin E2 (PGE2), giving them the ability to regulate immune responses. Furthermore, fibroblasts produce several growth factors as for example platelet-derived growth factor, transforming growth factor-β and insulin-like growth factors that regulate the reparative response, possibly involving autocrine regulatory loops (58). Finally immune functions of fibroblast may include interaction with myeloid cells, lymphocyte mobilization as well as induction of pro-inflammatory attributes, as observed in inflammatory disorders and cancer (59). Although fibroblasts may take part in immune responses in these diseases, it remains largely unexplored whether and what immune cell functions they may exert in the heart.

### Endothelial Cells – An Underrecognized Force

In the heart, endothelial cells cover the inner lumen of cardiac chambers, blood, and lymphatic vessels. Using immunohistochemistry, it has been estimated that endothelial cells cover more than 60% of non-myocyte cardiac cells in the adult mouse heart (60). Endothelial cells (ECs) fulfill a number of different tasks, including control of blood flow by modulating the degree of vascular relaxation and constriction, regulation of extravasation of solutes, fluid, macromolecules and hormones, participation in leukocyte trafficking and hemostasis, and contributions to thermoregulation and angiogenesis (61, 62). Based on the multitude of different functions within a variety of distinct tissues, endothelial cells show a high degree of heterogeneity, are equipped with specific properties, and exert different morphological features, all regulated by differences in the cellular gene expression programs (63, 64).

Despite the cellular heterogeneity of endothelial cells, markers could be identified to distinguish this cell type from other cardiac cells. Endothelial cells of the adult human heart can be identified by platelet and endothelial cell adhesion molecule 1 (*PECAM1*), cadherin 5 (*CDH5*), and von Willebrand factor (*VWF*), and subdivision into arterial endothelial cells is possible by prospero homeobox 1 (*PROX1*), FMS related tyrosine kinase 4 (*FLT4*), podoplanin (*PDPN*), B one M arrow tyrosine kinase gene in chromosome X non-receptor tyrosine kinase (*BMX*), and natriuretic peptide receptor 3 (*NPR3*) (16). In mice, *NPR3* is selectively expressed in adult endocardium (65). In the fetal human heart, endothelial cells can be identified using Sry-type box transcription factor (*SOX*) 7, 17, and 18, *PECAM*1, and *CDH5*. It is important to mention that these cells can undergo endothelial-mesenchymal transition and will then express genes that have been mostly used to identify fibroblasts (*COL3A1*; *COL1A2*; fibronectin 1, *FN1*; and biglycan, *BGN*). Thus, a deeper characterization of these cells is highly recommended. Other genes that are useful for the identification of endothelial cells are apolipoprotein E (*APOE*), intercellular adhesion molecule 2 (*ICAM2*), tyrosine kinase with immunoglobulin like and EGF-like domains 2 (*TIE2*), endoglin (*ENG*), and nitric oxide synthase 1 and 2 (*NOS1* and *NOS2*) (15, 66). Further classification between venous and arterial endothelial cells is feasible with the following genes: EPH receptor B4 (*EPHB4*), neuropilin 1 and 2 (*NRP1* and *NRP2*), nuclear receptor subfamily 2 group F member 2 (*NR2F2*), ephrin B1 (*EFNB*), delta like canonical Notch ligand 4 (*DLL4*) and *HEY1/2* (61). Lymphatic endothelial cells are found in a low percentage in the heart, and these are expressing *PROX1*, lymphatic vessel endothelial hyaluronan receptor 1 (*LYVEL*), *FLT4*, and *PDPN* (60). Interestingly, it was observed that endothelial cells are also able to switch on cardiomyocyte lineage genes such as *MYL2*, myoglobin (*MB*), *MYL3*, *TNNT2*, *TNNI3*, and ACTC1 following myocardial infarction, indicating the utility of transcriptional profiling and cell marker analysis in detecting cell type shifts, thus facilitating understanding of pathology, e.g., a cell type shift from endothelial cell phenotype to cardiomyocyte phenotype following myocardial infarction (67).

Some genes related to the role of endothelial cells in vascular tension, permeability and vessel formation are differentially expressed in young and adult mice. In healthy, 10-day-old mice, genes such as cytochrome c oxidase subunit 6A2 (*COX6A2*), cardiac myosin binding protein C (*MYBPC3*), myosin heavy chain associated RNA transcript (*MHRT*), *NPR3*, *TIE1*, and *TIE2* are clearly expressed (47). However, endothelial cells in adult mouse hearts express *CDH5*, *PECAM1*, fatty acid binding protein 4 (*FABP4*), *VWF*, and VCAM1 (48, 68–70). It is important to mention that although these markers are very specific for endothelial cells, other genes *GJA1*; ATPase sarcoplasmic/endoplasmic reticulum calcium transporting 2, *ATP2A2*; *TTN*; *RYR2*; *MYH6*) have also been reported that are related to cardiomyocyte function (71). Once cardiac tissue suffers a damage due to myocardial infarction, e.g., 3 days following coronary artery ligation, genes involved in leukocyte migration [e.g., chemokine (C–C motif) ligand 9 (*CCL9*), C–X–C motif chemokine ligand 2 (*CXCL2*)] are upregulated in endothelial cells. Intriguingly, different genes related to collagen production (*COLl3A1*), ribosome assembly and protein translation [ribosomal protein L9 (*RPL9*) and S12 (*RPS12*)], and cell proliferation [tumor protein, translationally controlled 1 (*TPT1*)], are enriched in endothelial cells 7 days following myocardial infarction (72). In heart failure, the most relevant genes upregulated in endothelial cells are mainly related to cell adhesion, angiogenesis, and cell migration (major histocompatibility complex, class I, B, *HLA-B*; EGF like domain multiple 7, *EGFL7*; receptor activity modifying protein 1 and 2, *RAMP1, RAMP2*; plasmalemma vesicle associated protein, *PLVAP*; inhibitor of DNA binding 1, *ID1*; and formin like, *FMNL3*), inflammatory response (*CX3CL1*; cluster of differentiation 74, *CD74*), as well as development and maturation (*SOX17, SOX18*) (73).

### Immune Cells – Regulators of Health and Disease in the Heart

The more frequent use of single-cell immune profiling in combination with advanced visualization technologies has profoundly deepened our understanding of the immune system of the heart, revealing the presence of a diverse landscape of innate and adaptive immune cells. Virtually all known types of immune cells have been described within the heart of both human and rodents including monocytes/macrophages, T-cells, B-cells, natural killer (NK) cells, and mast cells. Their precise roles often are yet to be fully defined (74). A recent study showed monocytes constituted 4.3% of all cells within fetal human hearts. They were identified by expression of basic leucine zipper ATF-like transcription factor 3 (*BATF3*), lysozyme (*LYZ*), *S100A8* and *S100A6*. Macrophages accounted for approximately 4.7% of all cells within the fetal human heart. Proliferating macrophages expressed the markers membrane spanning 4-domains A4A (*MS4A4A*), selenoprotein P (*SEPP1*), and *CD68*, while non-proliferating macrophages expressed additionally *MKI67*, *LYZ*, and *S100A6*. Around 5% of all cells were T cells, predominantly expressing GATA binding protein 3 (*GATA3*), lymphotoxin beta (*LTB*), and interleukin 7 receptor (*IL7R*), 2.4% of all cells were NK cells and expressed eomesodermin (*EOMES*), natural killer cell granule protein 7 (*NKG7*), granulysin (GNLY), granzyme A (*GZMA*), granzyme B (*GZMB*), and perforin 1 (*PRF1*). B cells represented 3.2% of all cells with signatures showing expression of B cell CLL/lymphoma 11A (*BCL11A*), membrane spanning 4-domains A1 (MS4A1), and immunoglobulin lambda like polypeptide 5 (*IGLL5*). Mast cells (1.3% all cells) could be separated by expression of tryptase beta 2 (*TPSB2*) and *GATA2* and dendritic cells by that of *CD1C*^+^, respectively (15).

Macrophages can be commonly classified by their phenotype and function into M1 and M2 polarized macrophages. Classically activated macrophages (M1 polarization) express interleukin 1β (*IL1B*), *CCL2*, *CCL9*, CXCL3, and usually govern pro-inflammatory functions (16, 75). In contrast, M2 polarized, non-classical macrophages are more likely to express *APOE*, galectin-3 (*LGALS3*) and the transmembrane glycoprotein NMB (*GPNMB*) and largely contribute to resolution of inflammation and repair (75).

As in fetal human hearts, myeloid cells represent the most prominent cellular fraction in adult human hearts. Commonly they are classified according to their C-C-chemokine receptor type 2 (*CCR2*) expression status into locally proliferating, self-renewing tissue resident (TR) *CCR2*^–^ macrophages, originally populating the heart from the yolk sac in early stages of embryonic development, and *CCR2*^+^ tissue resident macrophages (TRMs) stemming from the monocyte blood pool. While *CCR2*^–^ TRMs are abundant in the healthy state and instrumental for repair following damage, e.g. after myocardial infarction (MI), *CCR2*^+^ TRMs are rare in healthy states but quickly recruited upon injury and frequently mediating disease (76). The latter are not only recruited from blood and bone marrow but also from other tissues functioning as a reservoir to ensure timely recruitment during onset of inflammation (77, 78). Combining genetic fate mapping with scRNAseq, Dick et al. identified three clusters of TRMs: T cell immunoglobulin and mucin domain containing 4 positive, *LYVE1* positive, major histocompatibility complex class II low and *CCR2* negative (*TIMD4^+^LYVE1^+^MHC-II*^lo^*CCR2*^–^) relying almost exclusively on self-renewing by proliferation, *TIMD4^–^LYVE1^+^MHC^–^II*^hi^*CCR2*^–^ that are partially renewed from blood monocytes and finally *TIMD4^–^LYVE1^–^MHCII*^hi^*CCR2*^+^ that recruit themselves from exterior monocytes only. Interestingly, the TIMD4^+^CCR2^–^ group limited adverse remodeling in a mouse model of MI (79). TRMs have not only been implicated with myocardial infarction but with various other cardiac pathologies including myocarditis. Here, macrophages expressing mast cell immunoglobulin like receptor 1 (*MILR1*), *CXCL9*, lymphocyte antigen 6 complex, locus I (*LY6I*), *NOS2*, arginase 1 (*ARG1*), argininosuccinate synthase 1 (*ASS1*) appeared to entertain the inflammatory process (66). In addition, cardiac hypertrophy mimicked by the transverse aortic constriction has been linked to proinflammatory TRMs expressing Oncostatin M (80, 81). Important note has been found a correlation of human expression genes and mouse, *CCR2*^+^ macrophage abundance is associated with left ventricle (LV) remodeling and systolic function in heart failure patients (77).

Macrophage gene signature characteristically differs between male and female human hearts. Male cardiac macrophages upregulate genes involved in responding to foreign antigens, antigen processing, and presentation via MHC class II molecules such as interferon regulatory factor 8 (*IRF8*), a gene linked to chronic inflammation (82). In contrast, female-upregulated genes in cardiac macrophages are involved in the response to stress and the electron transport chain, e.g., the TSC22 domain family member 3 (*TSC22D3*, also known as Gilz), the most upregulated gene in female macrophages and the most sexually dimorphic macrophage gene between both sexes (68), a transcription factor implicated in anti-inflammatory functions and a downstream driver of the potent anti-inflammatory effects of glucocorticoids (83–85).

Constructing a cell atlas of the human heart from scRNAseq data, Tucker et al. identified two main immunologic cell clusters: A) a cluster representing TRMs expressing the scavenger receptors *CD163* and collectin subfamily member 12 (*COLEC12*), the mannose receptor C-type 1 (*MRC1*), the E3 ubiquitin ligase membrane associated ring-CH-type finger 1 (*MARCH1*), and the natural resistance-associated macrophage protein 1 (*NRAMP1*) which could be further separated into two macrophage clusters, both M2-like, expressing recombination signal binding protein for immunoglobulin kappa J region (*RBPJ*) and coagulation factor XIII A chain (*F13A1*) on the one hand and the transmembrane collagen *COL23A1* on the other (16). B) an immune cell cluster showing a T cell phenotype expressing the T cell surface antigen *CD2*, the early T cell antigen *CD69*, and the T-cell receptor-associated transmembrane adaptor 1 (*TRAT1*), the T cell immune adaptor src kinase associated phosphoprotein 1 (*SKAP1*), and the thymocyte selection marker *CD53* (16).

Immune cells of the adaptive immune system are the second largest cell fraction within the human heart and have been implicated with various cardiovascular diseases including myocardial infarction, myocarditis, and heart failure summarized elsewhere in a recent review by Steffens et al. (74). For example, T regulatory cells (Tregs) showing elevated expression of forkhead box P3 (*FOXP3)*, *CD25*, cytotoxic T-lymphocyte associated protein 4 (*CTLA4)*, and killer cell lectin like receptor G1 (*KLRG1)* were identified during MI in mouse hearts (86). However, interferon gamma (*IFNG*), tumor necrosis factor (*TNF*), IL3 and IL17 genes, related to classically polarized Th cells were not differentially expressed after MI in mice (87). In the same model, B cells presented with upregulation of activation markers such as *CD69*, *CCR7*, CXC-chemokine receptor type 5 (*CXCR5*), and transforming growth factor beta 1 (*TGFB1*) (88).

Other immune cell types found in the adult human heart include granulocytes expressing *CCR1*, colony stimulating factor 3 receptor (*CSF3R*), and *S100A9*, B-cells expressing *MS4A1* (14) and dendritic cells that – despite their very low number in cardiac tissue – may be identified by expression of *CD209a* (16). The knowledge about their significance for cardiac diseases is still very limited.

### Other Cell Types – Rare and Underrecognized

Recently, it has become evident that the cellular diversity of the heart stretches beyond cardiomyocytes, endothelial cells, fibroblast, and immune cells. Some of the more untraditional cells found in the heart are:

#### Adipocytes

Epicardial adipose tissue covers up to 80% of a human heart while it is essentially absent in rodents (89). Therefore, adipocytes account for up to 20% of the total mass (90). Since epicardial adipose tissue is supplied with blood thought the coronary circulation and it has a common embryonic origin with the heart, it has been suggested that it might be important for cardiac physiology (89). Epicardial adipose tissue can be segregated into adipocytes, preadipocytes and the so-called stroma vascular fraction comprising various cell types such as vascular cells and fibroblasts. Although the exact role of adipocytes remains unknown, it is possible that they might serve as a local energy store or to protect cardiomyocytes from lipotoxicity and hypothermia (91). Marker genes used for identification of adipocytes are those regulating the size and lipid droplet stability, including cell death-inducing DNA fragmentation factor, alpha subunit like effector c (*CIDEC*) and perilipin 5 (*PLIN5*). Furthermore, cardiac adipocytes are notably enriched of adiponectin, C1Q and collagen domain containing (*ADIPOQ*), which plays a role in the regulation of fatty acid transport and intracellular calcium homeostasis, as well as in thyrotropin releasing hormone degrading enzyme (*TRHDE*) expression, a gene responsible for inactivation of the thyrotropin release hormone.

Surprisingly, these cells also overexpress *IGF1* and T cell-activated increased late expression (*TACTILE* also known as *CD96*), markers involved in cell growth and proliferation in different cell types (16). While the mentioned studies are supportive of adipocytes playing a role in the development of CVD, descriptive and mechanistic studies are scarce in this topic.

#### Pericytes/Smooth Muscle Cells

The main difference between pericytes and smooth muscle cells is that pericytes reside within micro vessels, whereas smooth muscle cells contribute to the vascular wall of larger vessels. In human hearts, pericytes are characterized by genetic expression of platelet-derived growth factor receptor beta (*PDFRB*), ATP binding cassette subfamily C member 9 (*ABCC9*), and potassium voltage-gated channel subfamily J member 8 (*KCNJ8*), and it is possible to subdivide them by the expression of some adhesion molecules such as neural cell adhesion molecule 2 (*NCAM2*) and *CD38*, or with a gene related in microvascular morphogenesis, chondroitin sulfate proteoglycan 4 (*CSPG4*). Expression of *MYH11*, known as smooth muscle actin, generates a debate on the specificity of this gene (14). A recent study provided additional evidence on reliable genetic tools that can be used to identify, label, and target cardiac pericytes in mice, thereby facilitating further investigation of the role of this understudied cell type in heart disease (92).

Proteins related with contractile function such as *MYH11*, *ACTA2*, *TAGLN*, *RGS5*, vitronectin (*VTN*), *KCNJ8*, and myocardin (*MYOCD*) are used to identify smooth muscle cells (48, 68, 72). Until now, no changes in smooth muscle cells have been reported in the heart during any CVD, but one study performed in smooth muscle cells of ascending aortic wall in patients with myocardial infarction showed that at least 21 genes were upregulated in comparison with the control group (non-myocardial infarction patients). Those genes were related to three different functions such as the regulation of smooth muscle cell contraction by ATPase Na^+^/K^+^ transporting subunit alpha 2 (*ATP1A2*), superoxide dismutase 1 (*SOD1*), and *MYOCD*, heart development by histone deacetylase 9 (*HDAC9*), polycystin 2, transient receptor potential cation channel (*PKD2*), hexamethylene bisacetamide inducible 1 (*HEXIM1*), *FOXP1*, and integrin subunit beta 1 (*ITGB1*), and not less important actin cytoskeleton organization by spectrin alpha, erythrocytic 1 (*SPTA1*), platelet activating factor acetylhydrolase 1b regulatory subunit 1 (*PAFAH1B1*), erythrocyte membrane protein band 4.1 like 2 (*EPB41L2*), and profilin 1 (*PFN1*) (93). This shows that these cells may play an important role in the development of CVD, but so far, they have not been identified.

#### Mesothelial Cells

The mesothelial layer covering the heart has a crucial role in cardiac development and repair after injury. The most recent insights into cellular composition and diversity of the epicardium have lately been summarized comprehensively (94). This distinct small population of mesothelial cells expresses Wilms tumor 1 (*WT1*), basonuclin 1 (*BNC1*), basonuclin 2 (*BNC2*), and odd-skipped related transcription factor 1 (*OSR1*) under normal conditions, while neuropeptide Y (*NPY*) has been described to be responsible for cardiac remodeling, angiogenesis and vasoconstriction (14, 16). This cell subpopulation also expresses unspecific genes such as slow muscle troponin T1, slow skeletal type (*TNNT1*) or genes involved in immune response (complement C1r, *C1R*; complement factor I, *CFI*; complement C3, C*3*; and serpin family G member 1, *SERPING1*) (16).

#### Glia Cells/Schwann Cells

This cell type covers all surfaces of neuronal cells, and it has been shown to regulate tissue remodeling in a paracrine fashion (95). Peripheral glial cells can be separated into two main types; satellite glial cells covering neuron cell bodies located in ganglia, and Schwann cells which wrap nerve fibers. Since cardiac glial cells are dispersed throughout the heart, they were particularly difficult to analyze until the development of the scRNAseq methodology. However, scRNAseq studies have not yet examined sufficient cardiac glial cells to either detect subpopulations or investigate their change during heart development and disease. Although these cells are confirmed to be present in the heart (16), approaches to isolate these cells are limited. Generally used markers include *CSPG4* [also called Nerve/glial antigen 2 (*NG2*)], *PDGFRB*, and melanoma cell adhesion molecule (*MCAM*), which are non-specific; *CSPG4* and *MCAM* are expressed as well in mural cells, and *PDGFRB* expression is found in mural cells and fibroblasts. It is thought that *CD59a* might be a good marker (68), but this marker is also highly expressed in endothelial cells.

#### Progenitor/Progenerative Cells

Using radiocarbon (^14^C) birth dating and design-based stereology Bergmann et al. provided compelling evidence for the strikingly low regeneration capacity of human cardiomyocytes, with less than 1% renewing yearly in an adult human heart (13). Nevertheless, identification and understanding of progenitor/progenerative cells is highly relevant, as unlocking regenerative potential of contractile cells could provide means to rescue an injured heart (96). Over the last decade, two potential sources of cardiomyocyte renewal were extensively studied—pre-existing cardiomyocytes undergoing dedifferentiation and duplication, and stem or progenitor cells that contribute to *de novo* generation of cardiomyocytes—with recent work (97, 98) and subsequent consensus (99) favoring the former. By combining genetic fate-mapping with stable isotope labeling and multi-isotope imaging mass spectrometry, it was demonstrated that cardiomyocyte turnover in adult heart is primarily driven by the division of pre-existing cardiomyocytes during normal aging and after myocardial injury (98). Furthermore, a population of cardiomyocytes with a high pro-regenerative profile was identified in infant patients with dilated cardiomyopathy but was absent in children >6 years of age (100). In addition, an integrative cluster analysis of adult murine hearts obtained from multiple data sets discovered a minor population of cardiomyocytes characterized by proliferation markers that could not be identified by analyzing the datasets individually (101), further supporting the idea that the renewal of the cardiomyocyte pool is driven by cytokines of resident cardiomyocytes rather than differentiation of progenitor cells.

On the other hand, cardiac progenitor cells are made up of different cell types characterized by the expression of proto-oncogene receptor tyrosine kinase (*KIT*), *LY6A*, ATP binding cassette subfamily G member 2 (*ABCG2*), *ISL1*, and *TBX18* (25, 100–103). However, recent genetic lineage tracing studies revealing that only a small fraction of endogenous cells expressing *LY6A* or *KIT* contribute to the adult cardiomyocyte population challenge the view that newly formed cardiomyocytes are predominantly derived from cardiac progenitor cells (69, 104, 105). Even with these data, it has not yet been possible to define whether the cardiomyocyte renewal, if any, is originated by the generation of new cardiomyocytes from a rare division of existing cardiomyocytes or from putative cardiac stem cells after cardiac injury (96, 106).

NKX2 Homeobox (*NKX2*) and *ISL1* expression has led to the identification of previously unknown progenitor subpopulations during the early phase of cardiac fate decision-making (107). In addition, a population of cardiomyocytes with a high pro-regenerative profile was identified in infant patients with dilated cardiomyopathy but was absent in children >6 years of age (108). It is broadly accepted that adult heart also has an, albeit very limited, regenerative potential. Its origin, however, is still matter of an ongoing debate. An integrative cluster analysis of adult murine hearts obtained from multiple data sets discovered a minor population of cardiomyocytes characterized by proliferation markers that could not be identified by analyzing the datasets individually (97).

It is now widely accepted that the heart has an, albeit very restricted, regenerative potential. However, further work in needed to identify and characterize populations of proliferative cardiomyocytes and mechanisms of endogenous renewal that could be exploited for repairing the injured myocardium.

## Discussion

In recent years, scRNAseq has made a quantum leap from large-scale cell population studies to single cell analysis. Despite its short history, scRNAseq has already begun to drive new discoveries in different disciplines that would not have been possible with traditional methods such as for example FACS analysis. International collaborative efforts of multiple laboratories aim to define the cellular heterogeneity in all organ systems. The Human Cell Atlas (109) and Human BioMolecular Atlas Program^1^ are of particular importance for human physiology and pathophysiology, while the Tabula Muris project (32) allows for deconvolution of murine single cell subtype transcriptome. In addition, Asp et al. combined scRNAseq data of human embryonic cardiac cells, RNA-seq data of spatial transcriptomics, and *in situ* sequencing data to map cell-type distribution and spatial organization in the human embryonic heart and generate a 3D gene profile atlas of the developing human heart (110). Using a similar approach to study the development of the chicken heart from the early to late four-chambered heart stage, Mantri et al. identified diverse cellular lineages in developing hearts, their spatial organization, and their interactions during development (111). Although the atlases generated from scRNAseq technology are becoming more and more complete, one of the crucial remaining tasks that will facilitate their effective integration into future clinical trials, is standardization of the markers used for the different cells, as well as experimental and analytical pipelines.

Despite challenges associated to tissue availability and cellular isolation (112), in the cardiovascular field alone, numerous groups have used scRNAseq technology to identify new cell populations and key molecular players driving numerous physiological and pathophysiological processes in the heart. Using scRNAseq of isolated cardiomyocytes from heart failure patients with ventricular arrhythmia, Yamaguchi et al. recently identified a subpopulation of cardiomyocytes which readily expresses dopamine D1 receptor (113). Following the lead from untargeted transcriptomic analyses, they further generated cardiomyocyte-specific D1 receptor knockout and overexpressing mice and proved that cardiac D1R receptor upregulation is both necessary and sufficient for inducing life-threatening ventricular arrhythmia.

scRNAseq of cells from a commonly used heart failure model—transverse aortic constriction (114) mouse model—facilitated novel discoveries with important clinical implications. Transcriptome analysis of >11,000 single cells revealed that activation of proinflammatory macrophages is the key event in the transition from normal to reduced ejection fraction (115). Furthermore, macrophage activation and subtype switching, a key event at middle-stage of cardiac hypertrophy, was effectively attenuated by Dapagliflozin, a sodium glucose cotransporter 2 inhibitor known for its beneficial effects in heart failure patients, as well as two additional anti-inflammatory agents, inhibitor of galectin-3 (TD139) and Arglabin, which are rarely used in setting of cardiac diseases. Importantly, the authors could confirm similar molecular and cellular patterns in human samples of hypertrophic cardiomyopathy and heart failure. Nomura et al. manually isolated single cardiomyocytes from wild-type and *p53* cardiomyocyte-specific deficient mice in the presence or absence of TAC (38). They subsequently analyzed transcriptomes of 473 cardiomyocytes and found that continuous pressure overload leads to a cardiomyocyte divergence into adaptive and failing phenotypes, and that p53 signaling is specifically responsible for alterations typical for late cardiac remodeling. Again, accompanying human single-cardiomyocyte analysis validated the conservation of the pathogenic transcriptional signatures in heart failure patients. Satoh et al. applied three single-cell analysis methods, namely, sc-qPCR, scRNAseq, and single-molecule fluorescence *in situ* hybridization (smFISH) to study transcriptome profile in isolated cardiomyocytes and cross sections from TAC murine hearts at an early hypertrophy stage (2 weeks post-TAC) and at a late heart failure stage (8 weeks post-TAC) (116). In alignment with the idea of cardiomyocytes progressing into different phenotypes over the course of the remodeling, expression levels of *MYH7*, a representative fetal gene, greatly varied in hypertrophic cardiomyocytes and was more consistently found in failing cardiomyocytes. *MYH7*-expressing cardiomyocytes were significantly more abundant in the middle layer, compared with the inner or outer layers of hypertrophic hearts, while such spatial differences were not observed in failing hearts. Interestingly, expression of *MYH7* was negatively correlated with cellular size and abundance of mitochondria-related gene transcripts.

In a rat model of heart failure with preserved ejection fraction, scRNAseq transcriptome analyses of the sinoatrial node revealed significant alterations in both the “membrane clock” (ion channels) and the “calcium clock” (spontaneous calcium release events) which—when probed in functional experiments—further validated RNA-seq data (117).

Dong et al. performed meta-analyses of large-scale, publicly available bulk and single-cell RNA sequencing datasets to identify vascular smooth muscle cell (VSMC)-enriched long non-coding RNAs. The role of novel VSMC-expressed long non-coding RNA, cardiac mesoderm enhancer-associated non-coding RNA (*CARMN*), was then investigated in VSMC-specific *CARMN* knockout mice that underwent carotid artery injury. *In vivo*, *CARMN* deletion in VSMC exacerbated, while its overexpression markedly attenuated injury-induced neointima formation in two independent animal models, underscoring its potential clinical implication as a therapeutic target for intimal hyperplasia (80).

Although there are only a few reports on spatial transcriptomics in the heart, two areas of cardiac research are particularly dependent on detailed understanding of the spatial transcriptome patterns. First, cardiac development is a spatially complex process and comprehensive understanding of regional changes in gene expression during heart maturation is of great interest. Second, spatial information is crucial in myocardial infarction, given that localized occlusion of a coronary artery differentially affects the site directly adjacent to the infarct site, whereas the remote areas are only indirectly affected. Accordingly, care must be taken when developing treatment strategies. In conditions where only a portion of cells shows alterations in signaling pathways, we must learn more about the specific cell type, their localization in the heart, as well as the temporal resolution of their reprogramming in order to introduce the treatment, when and where this trigger is detrimental, and to reduce off-target effects.

Two important factors complicate interpretation of data on cellular heterogeneity of the heart. First, there are continuous fluctuations in abundance of diverse cellular lineages, their spatial organization and molecular composition, as well as their interactions during heart development. Second, well-documented inter-species differences must be considered when extrapolating data from experimental animal models to human cardiac physiology and pathophysiology. Therefore, the high-level standardization of markers for different cell types, their developmental stage and their host species is crucial for the comprehensive understanding of cellular heterogeneity in cardiac health and disease.

Overall, our knowledge on the cellular composition and its dynamic changes in cardiac health and disease is steadily increasing through the advent of powerful technologies such as scRNAseq. scRNAseq technology alone and particularly in combination with spatio-temporal genetic and/or proteomic data has a potential to transform our knowledge on disease mechanisms, more precisely predict patients at risk of developing adverse cardiac outcomes and reveal mechanisms underlying distinct personalized therapeutic responses. It, therefore, holds a promise to become an integral and central part of future clinical trials (118).

## Author Contributions

NA and AZ conceptualized the manuscript. NA, SL-H, HB, and AZ contributed to the research for writing the manuscript. NA and SL-H designed the figure and tables. All authors contributed to the discussion, writing, and review of the manuscript.

## Conflict of Interest

The authors declare that the research was conducted in the absence of any commercial or financial relationships that could be construed as a potential conflict of interest.

## Publisher’s Note

All claims expressed in this article are solely those of the authors and do not necessarily represent those of their affiliated organizations, or those of the publisher, the editors and the reviewers. Any product that may be evaluated in this article, or claim that may be made by its manufacturer, is not guaranteed or endorsed by the publisher.

## Footnotes

1
https://commonfund.nih.gov/hubmap
