# Supplementary material for: Cellular Heterogeneity of the Heart
Source: Front Cardiovasc Med. 2022 Apr 25;9:868466. doi: 10.3389/fcvm.2022.868466 (PMC9081371; doi:10.3389/fcvm.2022.868466)
Supplement: Supplementary file 1 [file Data_Sheet_1.docx]

**Supplementary Table 1**. Markers that may be used to identify different cell types in the mouse heart.

| **Cell Type (age)** | **Genes** | **Reference** |
| --- | --- | --- |
| Cardiomyocytes  (fetal) | MYH6, MYH7, TBX3, SMOC2, IGFBP5, CPNE5, NTM | Sassoon, Garner et al. 1988, Hoogaars, et al. 2004, van Eif et al, 2019, Goodyer, et al. 2019, |
| Endothelial cells  (10 days postnatal) | COX6A2, MYBPC3, MHRT, NPR3, TIE1, TIE2 | Hu P, et al. 2018 |
| Cardiomyocytes  (adult) | ACTN2, TNNC1, ACTC1, RYR2, MYH6, ACTC1, TNNT2, NPPA, MYL7, MYH6, TTN, MYH7, HEY2, TPM1, TTN, GJA1, MYOZ2, TTN, MYBPC3, TNNT2, MYH7, MYL2, HCN4, CNTN2, MYOZ2 | Galow et al., 2020, Kretzschmar et al., 2018, Litviňuková et al., 2020, Wolfien et al. 2020, Skelly D, et al. 2018, Gladka, et al. 2018, van Eif, Stefanovic et al. 2019, Pallante B, et al. 2010, Liang X et al, 2013, |
| Endothelial cells  (adult) | PECAM1, NPR3, COL3A1, COL1A2, FN1, BGN, APOE, ICAM2, TIE2, ENG, NOS1, NOS2, EPHB4, NRP1, NRP2, NR2F2, EFNB, DLL4, HEY1, HEY2PROX1, LYVEL, FLTA, PDPN, MYL2, MB, MYL3, TNNT2, TNNI3, ACTC1, CDH5, VWF, FABP4, VCAM1, 1GJA1, ATP2A2, TTN, RYR2, MYH6, CCL9, CXCL2, COL3A1, RPL9, RPS12, TPT1 | Galow et. Al. 2020, McLellan et al., 2020, Wolfien et al.2020, Kalucka J, et al. 2020, Forte E, et al. 2020, Zhuang L, et al. 2020, Skelly D, et al. 2018, Air W et al. 2007, Pinto A et al. 2016, Hua, X et al. 2020, Li Z, et al. 2019, van Berlo J, et al. 2014, Kalucka J et al. 2020, Zhuang L, et al. 2020, |
| Fibroblasts  (adult) | POSTN, FN1, CKAP4, COL1A1, COL1A2, COL6A1, PDGFRA, PDGFRB, VTN, DCN, GSN, TAGLN, RGS5, ACTA2, CD90.1, PDGFRA, S100A4, DDR2, SCA1, VIMENTIN, COL5A1, PERIOSTIN, aSMA, CILP, ITGBL1, CKAP4, NOX4, IGF1, ADAMTS4, VCAM, AXL, POSTN, WISP1, TNC, MT2, DKK3, TCF21, FBN1, KI67, SPARC, SFRP2, DRR2, RUNX2, GPM6B, JUNB, CEBPB, CTHRC1, FAP | Gladka et al., 2018, Kretzschmar et al., 2018, McLellan et al., 2020, Zhuang L, et al. 2020, Skelly D, et al. 2018, Marin-Sedeno E et al. 2021, Tallquist M et al. 2017, Vistnes M et al. 2014, Battle M et al. 2019, Hu P et al. 2018, Kretzschmar K et al. 2018, Vidal R et al. 2019, Aghajanian et al. 2019 |
| Macrophages  (adult) | FCGR1, CSF1R, CCL2, CCL9, CXCL3, APOE, LGALS3, GPNMB, TIMD4, LYVE, MHCII, CXCL9, LY6I, NOS2, ARG1, ASS1, TSC22d3, | Molenaar B, et al. 2021, Dick S, et al. 2019, |
| T cells  (adult) | FOXP3, CD25, CTLA4, KLRG1 | Xia et, al, 2020 |
| Smooth muscle cells  (adult) | MYH11, ACTA2, TAGLN, RGS5, VTN, KCNJ8, MYOCD | Skelly D et al. 2018, Forte E et al. 2020, Zhuang L et al. 2020 |
| Schwann cells  (adult) | CSPG4, NG2, PDGFRB, MCAM, CD59a | Skelly D. A. et al, 2018. |

**Supplementary Table 2**. Markers that may be used to identify different cell types in the adult human heart.

| **Cell Type** | **Genes** | **Reference** |
| --- | --- | --- |
| Cardiomyocytes | MYL2-4, MYL7, MYL9, MYH6, MYH7, MYH7B, MYL2, MYBPC3, TNNT2, TNNTI1, TNNTI3, TNNTC1, RYR2, PLN, 1SLC8A1, PCDH7, SET, SMYD2, TTN, ACTC1, TOP2A, MKI67, HEY2, NPPA, PITX2, ANP, BNP, | Litviňuková et al., 2020, Tucker et al., 2020, Wang et al., 2020, Schwartz K, et al 1992, Boheler et al. 1992, Gugbjartsson et al. 2007, Razeghi et al. 2001, Dirkx et al. 2013, |
| Endothelial cells | PECAM1, CDH5, VWF, CX3CL1, CCL2, IL6, ICAM1, OSMR, ILST6, PROX1, FLT4, PDPN, PDGFRA, NPR3, HLA-B, EGFL7, RAMP1, RAMP2, PLVAP, FMNL3, CX3CL1, CD74, SOX17, SOX18, | Litviňuková et al., 2020, Tucker et al., 2020, Wang et al., 2020, Shi X, et al. 2021, |
| Pericytes | PDRFB, ABCC9, KCNJ8, NCAM2, CD38, CSPG4, MYH11, | Litviňuková et al., 2020 |
| Smooth muscle cells | AT1A2, SOD1, MYOCD, HDAC9, PKD2, HEXIM1, FOXP1, ITGB1, SPTA1, PAFAH1B1, EPB41L, PFN1 | Wongsurawat T t al. 2018 |
| Mesothelial cells | WT1, BNC1, BNC2, OSR1, CFI, C3, SERPING1, TNNT1, C1R | Litviňuková et al., 2020, Tucker et al., 2020 |
| Fibroblasts | POSTN, TNC, FN1, COL4A1, TNC, MYH11, DCN, GSN, TAGLN, RGS5, ACTA2, CD90.1, PDGFRA, S100A4, DDR2, SCA1, VIMENTIN, COL1A1, PERIOSTIN, aSMA, CILP, ITGBL1, CKAP4, NOX4, IGF1, ADAMTS4, VCAM, AXL, CTHRC1, AEBP1, MEOX1, FAP | Litviňuková et al., 2020, Tucker et al., 2020, Marin-Sedeno E et al. 2021, Tallquist M et al. 2017, Ruiz-Villalba et al. 2020, Rao M et al. 2021, Alexanian et al 2021, Tillmanns J et al. 2015, Aghajanian et al. 2019 |
| Macrophages | LYVE1, MRC1, NRAMP1, SLC11A1, HLA-DRA, HLA-DMA, HLA-DMB, HLA-DPA1, TREM2, CCL2, CCL9, CXCL3, COLEC12, MRC1, MARCH1, NRAMP1, RBPJ, F13A1, COL23A1, | Litviňuková et al., 2020, Tucker et al., 2020, Molenaar B, et al. 2021, Bjan Z et al, 2020, |
| Monocytes | LYVE1, FOLR2, CEBPB, S100A8, CCL13, CCL18 | Litviňuková et al., 2020 |
| Granulocytes | CCR1, CSF3R, S100A9, | Tucker et al., 2020 |
| Adipocytes | CD96, CIDEC, ADIPOQ, TRHDE, IGF1, PLIN5, TRHDE, C1Q | Litviňuková et al., 2020, Tucker et al., 2020 |
| B cells | MS4A1 | Tucker et al., 2020 |
| T cells | CD2, CD69, TRAT1, SKAP1, CD53, | Tucker et al., 2020 |
| Dendritic cells | CD209a | Tucker et al., 2020 |

**Supplementary Table 3**. Markers that may be used to identify different cell types in the fetal human heart.

| **Cell Type** | **Genes** | **Reference** |
| --- | --- | --- |
| Cardiomyocytes | MYL2-4, MYL7, MYL9, MYH6, MYH7, MYH7B, MYBPC3, TNNT2, TNNT2, TNNTI1, TNNTI3, TNNTC1, RYR2, PLN, 1SLC8A1, ACTA1, N2BA1/N2BA2, CPT-1, PPARa, PGC-1a, TBX3, SMOC2, MYOZ2 | Suryawanshi H, et at. 2020, Schwartz K, et al 1992, Lahmers, Wu et al. 2004, Taegtmeyer, Sen et al. 2010, van Eif et al, 2019, |
| T cells | GATA3, LTB, IL7R | Suryawanshi H, et at. 2020 |
| NK cells | OEMES, NKG7, GNLY, GZMA, GZMB, PRF1 | Suryawanshi H, et at. 2020 |
| B cells | BCL11A, MS4A1, IGLL5 | Suryawanshi H, et at. 2020 |
| Mast cells | TPSB2, GATA2 | Suryawanshi H, et at. 2020 |
| Dendritic cells | CD1c | Suryawanshi H, et at. 2020 |
| Myeloid cells | MS4A4A, SEPP1, CD68, LYZ, S100A8, S100A6 | Suryawanshi H, et at. 2020 |
| Endothelial cells | PECAM1, CDH5, SOX17, SOX18, COL3A1, COL1A2, FN1, BGN, APOE, ICAM2, TIE2, ENG, NOS1, NOS2 | Suryawanshi H, et at. 2020 |
| Fibroblasts | TCF21, SNAI2, COL1A1, COL1A2, DCN, DLK1, LUM, MEOX1 | Suryawanshi H, et at. 2020, Alexanian et al 2021 |
| Smooth muscle cells | DCN, DLK1, LUM, TAGLN, RGS5, ACTA2 | Suryawanshi H, et at. 2020 |
| Macrophages | MS4A4A, SEPP1, CD68, MKI67, LYZ, S100A6, CCR2 | Suryawanshi H, et at. 2020, Bjan Z et al, 2020, |
| Monocytes | BATF3, LYZ, S100A8, S100A6, | Suryawanshi H, et at. 2020 |
| Mesothelial cells | UPK3B, MSLN, WT1 | Suryawanshi H, et at. 2020 |
